# Differences in Immunological Landscape between *EGFR*-Mutated and Wild-Type Lung Adenocarcinoma

**DOI:** 10.1155/2021/3776854

**Published:** 2021-08-26

**Authors:** Jia-Wei Luo, Yan-Hua Guo, Feng-Ying Wu, Xue-Fei Li, Xue-Cheng Sun, Jia-Lu Wang, Cai-Cun Zhou

**Affiliations:** ^1^Department of Medical Oncology, Shanghai Pulmonary Hospital, Thoracic Cancer Institute, Tongji University School of Medicine, Shanghai 200433, China; ^2^Department of Thoracic Surgery, Tongji University Affiliated Shanghai Pulmonary Hospital, Shanghai 200433, China; ^3^Department of Pathology, Shanghai Tenth People's Hospital Affiliated to Tongji University, Shanghai 200040, China; ^4^Department of Pathology, East China Hospital Affiliated to Fudan University, Shanghai 200040, China

## Abstract

Recent clinical trials of lung adenocarcinoma with immune checkpoint inhibitors revealed that lung adenocarcinoma patients with *EGFR* mutations have a poor response to immunotherapy. However, the mechanisms have not been addressed. We performed immunohistochemistry analyses of resected lung adenocarcinoma tissues with and without *EGFR* mutations to investigate and compare the characteristics of the tumor microenvironment (TME). We retrospectively enrolled a total of 323 lung adenocarcinoma patients (164 had *EGFR* mutations), and their corresponding tissue samples were analyzed by the *EGFR* mutation test and immunohistochemistry. We selected the markers of the immune checkpoint molecule (PD1, PD-L1, and LAG-3) and immune cell (CD3, CD4, CD8, and Foxp3) as markers of the tumor microenvironment. Our results revealed that patients had a distinct tumor microenvironment between *EGFR*-mutant and wild-type lung adenocarcinomas; the expression of CD3, CD4, PD-L1, and Foxp3 in *EGFR*-mutant tumors was significantly higher than that in wild-type tumors, while the expression of LAG3 and PD-1 showed a positive correlation with *EGFR*-wild-type tumors. In survival analysis, *EGFR*-wild-type patients had longer disease-free survival (DFS) than *EGFR*-mutant patients (*P* = 0.0065). Our research demonstrates significant differences in tumor microenvironment composition between *EGFR*-mutant and wild-type patients. Our findings provide novel evidence that contributes to understanding the mechanism underlying the poor efficacy of immune checkpoint inhibitors.

## 1. Introduction

Immunotherapy targeting programmed cell death 1 (PD1) or its ligand, PD1 ligand 1 (PD-L1), has transformed the paradigm of lung cancer treatment. Durable clinical benefit was observed in advanced lung cancer patients treated with PD1/PD-L1 inhibitors [1, 2]. Accordingly, anti-PD1/PD-L1 treatment has been approved as a second-line or first-line treatment for advanced lung adenocarcinoma [3–5]. However, despite substantial achievements in clinical care, a considerable proportion of patients does not derive benefit from anti-PD1/PD-L1 treatment. Accumulating evidence has proved that patients with *EGFR* mutations cannot benefit from immunotherapy [6–8]. The underlying mechanism is still unclear. The PD-L1 expression level, tumor mutation burden (TMB), and infiltrated lymphocyte have been identified as predictive markers for anti-PD1/PD-L1 immunotherapy [9]. Several studies tried to find the mechanism of immune escape in this subgroup of patients. Unfortunately, still, no report could give us a satisfying answer.

Thus, in the present study, we investigated the expression of immune checkpoint inhibitors (PD1, PD-L1, and LAG3) and infiltration of immune cells (CD3^+^TILs, CD4^+^TILs, CD8^+^TILs, and Foxp3^+^Treg) in *EGFR*-mutated patients and matched wild-type patients, trying to elucidate immune landscape of *EGFR*-mutated lung cancer. Furthermore, we collect the survival of patients, analyzed the contribution of immune factors to the survival differences.

## 2. Materials and Methods

### 2.1. Patients

All patients in our study received surgical resection at Shanghai Huadong Hospital between January 2015 and December 2018. A total of 323 eligible patients were enrolled based on the following inclusion criteria: (i) they are pathologically diagnosed with lung adenocarcinoma, (ii) the pathological stage was IA to IIIA, (iii) the content of tumor tissue components can be observed on HE-stained sections ≥ 20%, and (iv) patient's clinical data was complete. Patient characteristics are summarized in Table 1. The median age was 68 years old (range 35–86 years) and 65.9% (*n* = 213) were men. The follow-up was completed on May 15^th^, 2020. The median follow-up was 21.8 months (ranging from 4 to 47 months). The investigation was approved by the scientific review and ethics committee of Shanghai Pulmonary Hospital.

### 2.2. Immunohistochemistry (IHC)

All specimens were examined by immunostaining tumor cells and TILs. Formalin-fixed paraffin-embedded xenograft tumors 3 *μ*m thick were dewaxed in xylene, hydrated in graded alcohols, and washed with PBS. After blocking endogenous peroxidase activity with 3% H_2_O_2_ aqueous solution for 10 minutes, the sections were incubated with primary antibodies overnight. After washing with PBS, they were then incubated with general-type IgG-HRP polymer for 10 minutes, followed by 3,3′-diaminobenzidine for about 3 minutes. Finally, the sections were retained with hematoxylin for 1 minute and then dehydrated in graded alcohols, cleared in xylene, and covered with coverslips [10]. Immunohistochemistry (IHC) was performed using the following antibodies: anti-human PD-L1 (clone E1L3N, Cell Signaling Technology, Danvers, MA, diluted 1 : 200), CD8 monoclonal antibodies (M7103, clone C8144B, DAKO, Glostrup, Denmark, diluted 1 : 200), CD3 monoclonal antibodies (DAKO, Santa Clara, CA, USA, diluted 1 : 200), CD4 monoclonal antibodies (Cell Marque, Rocklin, CA, USA, diluted 1 : 150), anti-Foxp3 antibody (236A/E7, ab20034, Abcam, Cambridge, USA, diluted 1 : 200), anti-Lag3 antibody (EPR20261, ab209236, Abcam, Cambridge, USA, diluted1:150), and anti-PD1 antibody (NAT105, ab52587, Abcam, Cambridge, USA, diluted 1 : 200). PD-L1 expression was evaluated based on the intensity and proportion of tumor cells displaying either in membranous or in cytoplasmic staining by two independent pathologist. Lymphocytes with the cytoplasmic expression of CD8 infiltrating within the tumor region, either in the central or in the marginal tumor region, were defined as CD8^+^TILs.

### 2.3. IHC Scoring Strategy

The markers were scored in four categories: no staining (0), weak staining (1, light-brown membrane staining, visible only with high magnification), intermediate staining (2, between 1 and 3), and strong staining (3, visible with low magnification, dark-brown linear membrane staining); according to the results, they are divided into high (score 2–3) and low (score 0–1) categories by two independent pathologists. As for PD-L1, a threshold of at least 5% of cells with membranous staining to define positivity was used.

### 2.4. *EGFR* Mutation Test

The *EGFR* status of all specimens mentioned before was carried out using amplification refractory mutation system-PCR (ARMS-PCR) technology on a Bio-Rad CFX96 machine (Bio-Rad, American), which is fully evaluated on histological tissue. The ADx *EGFR* Mutations Detection Kit (Amoy Diagnostics, China) was subjected to this procedure. There are 29 known mutations in exons 18–21 of *EGFR*, including G719X in exon 18, deletion in exon 19, S768I and T90M in exon 20, and L861Q in exon 21.

### 2.5. Statistical Analysis

The comparison of categorical variables and the association between various markers and clinicopathologic features were confirmed by the chi-square test or Fisher exact test, and the nonparametric test was used to evaluate the differences in continuous variables. The disease-free survival (DFS) was defined as the time from random assignment to the time of the first event (progression, death) [11]. Patients with no events were censored at the date of their last follow-up. The survival curve plots were completed by using the Kaplan–Meier method and compared by the log-rank test. Univariate analysis was used to identify the factors associated with DFS. All significant variables in univariate analysis were screened and enter the multivariate Cox regression analysis. All analyses were performed by IBM SPSS Statistics v 17.0 (IBM), and the *P* value of 0.05 was considered statistically significant.

## 3. Results

### 3.1. Clinicopathological Characteristics

The characteristics of patients are summarized in Table 1. A total of 323 early-stage lung adenocarcinoma patients (IA to IIIA) who received surgical treatment were included. Tissues from resected tumors were obtained. The median age at diagnosis was 68 years (ranging from 35 to 86). 213 patients (65.9%) were male and 32 (9.9%) patients had a history of smoking. Pathological TNM stages were I for 183 patients (56.7%), II for 99 patients (30.7%), and III for 50 patients (15.5%). 164 patients (50.8%) had *EGFR* mutations and 159 patients (49.2%) without *EGFR* mutation.

### 3.2. Expression of the Immune Checkpoint Molecule in *EGFR*-Mutant vs Wild-Type Lung Cancer Patients

**Figure 1:**
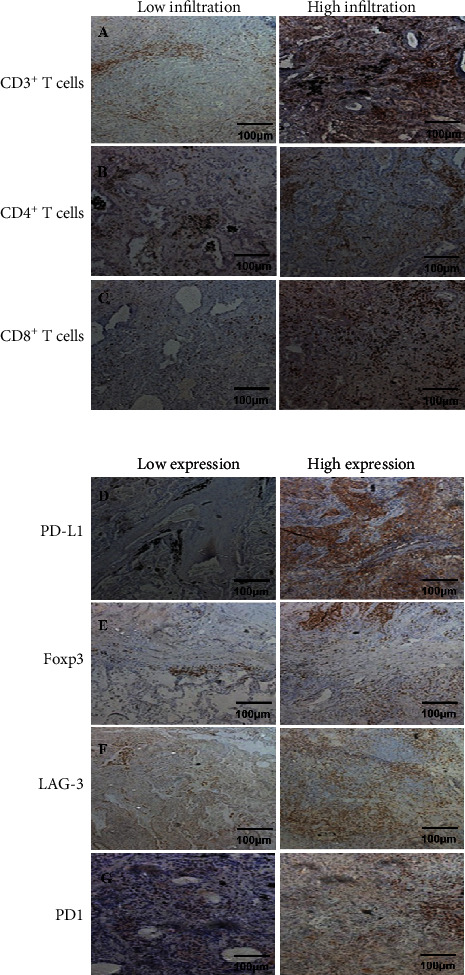
Schematic diagram of immunohistochemical staining of markers. (a) CD3^+^ T cell staining; (a), 1: low infiltration of CD3^+^ T cells; (a), 2: high infiltration of CD3^+^ T cells; (b) CD4^+^ T cell staining; (b), 1: low infiltration of CD4^+^ T cells; (b), 2: high infiltration of CD4^+^ T cells; (c) CD8^+^ T cell staining; (c), 1: low infiltration of CD8^+^ T cells; (c), 2: high infiltration of CD8^+^ T cells; (d) PD-L1 staining; (d), 1: low expression of PD-L1; (d), 2: high expression of PD-L1; (e) Foxp3 staining; (e), 1: low expression of Foxp3; (e), 2: high expression of Foxp3; (f) LAG-3 staining; (f), 1: low expression of LAG-3; (f), 2: high expression of LAG-3; (g) PD1 staining; (g), 1: low expression of PD1; (g), 2: high expression of PD1.

**Figure 2:**
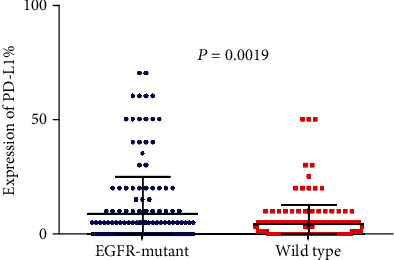
Comparison of PD-L1 expression between *EGFR*-mutant and wild-type lung adenocarcinoma tumor microenvironment.

**Figure 3:**
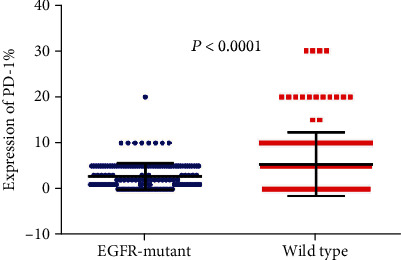
Comparison of PD-1 expression between *EGFR*-mutant and wild-type lung adenocarcinoma tumor microenvironment.

**Figure 4:**
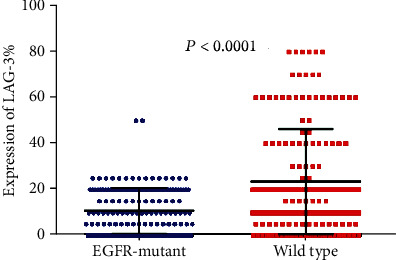
Comparison of LAG-3 expression between *EGFR*-mutant and wild-type lung adenocarcinoma tumor microenvironment.

To elucidate the expression of the immune checkpoint molecule in *EGFR*-mutated patients, we detect the expression of PD-L1, PD1, and LAG3 in all the samples (Figure 1). We found that PD-L1 expression is significantly higher in *EGFR*-mutated patients than in wild-type patients (*P* = 0.0019; Figure 2). However, the expression of PD-1 and LAG3 was lower in *EGFR*-mutated patients when compared with wild-type patients (*P* < 0.0001 vs *P* < 0.0001 separately; Figures 3 and 4).

### 3.3. Immune Cell Infiltration in *EGFR*-Mutated and Wild-Type Lung Cancer Patients

**Figure 5:**
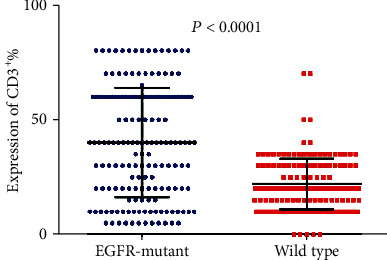
Comparison of CD3^+^ T cell infiltration between *EGFR*-mutant and wild-type lung adenocarcinoma tumor microenvironment.

**Figure 6:**
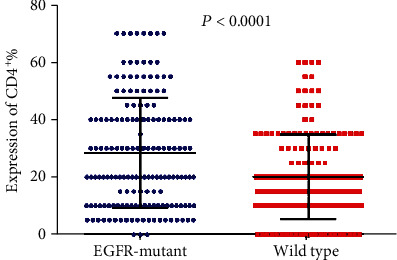
Comparison of CD4^+^ T cell infiltration between *EGFR*-mutant and wild-type lung adenocarcinoma tumor microenvironment.

**Figure 7:**
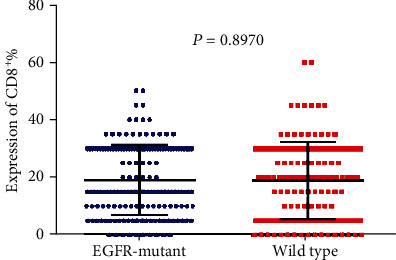
Comparison of CD8^+^ T cell infiltration between *EGFR*-mutant and wild-type lung adenocarcinoma tumor microenvironment,

**Figure 8:**
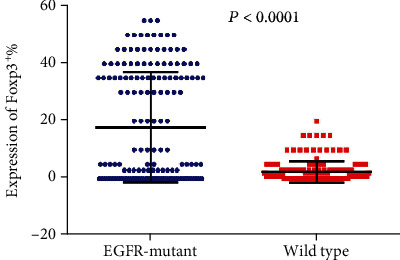
Comparison of Foxp3^+^ T cell infiltration between *EGFR*-mutant and wild-type lung adenocarcinoma tumor microenvironment.

Tumor-infiltrating lymphocytes (TILs) are an important predictive biomarker for anti-PD1 immunotherapy. Thus, we evaluated the infiltration of CD3^+^ T cell, CD4^+^ T cell, CD8^+^ T cell, and Foxp3^+^ T cell in *EGFR*-mutated and wild-type patients separately. We observed that the number of CD3^+^ TILs and CD4^+^ TILs is significantly higher in *EGFR*-mutant tumors than in wild-type tumors (*P* < 0.0001 and *P* < 0.0001 separately; Figures 5 and 6). However, no significant differences were observed for the infiltration of CD8^+^ TILs (*P* = 0.8970; Figure 7). Notably, the amounts of Foxp3^+^ TILs in the *EGFR*-mutant tumors were dramatically higher when compared with wild-type tumors (*P* < 0.0001; Figure 8).

### 3.4. The Outcome of Patients according to *EGFR* Mutation and the Immune Microenvironment

**Figure 9:**
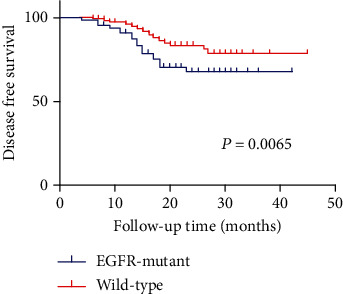
Comparison of postoperative DFS between *EGFR*-mutant and wild-type patients.

The gene mutation status and tumor microenvironment might be associated with the outcome of patients. We investigate the DFS of patients with different subgroups. As shown in the Kaplan–Meier curve, DFS was longer in the wild-type patients than in the *EGFR*-mutant patients (*P* = 0.0065; Figure 9). Moreover, we divided patients into 7 subgroups according to the immune microenvironment: PD-L1^+^/PD-L1^−^, CD3^high^/CD3^low^, CD4^high^/CD4^low^, CD8^high^/CD8^low^, Foxp3^high^/Foxp3^low^, LAG3^high^/LAG3^low^, and PD1^high^/PD1^low^.

Among the whole cohort, univariate and multivariate analyses were undertaken to find factors that contribute to DFS in *EGFR*-mutated and wild-type patients separately (Table 2). We found that age ≥ 66 years (age < 66 years; HR = 1.169; 95% CI,1.112–1.877; *P* = 0.009) and Foxp^low^ TILs (Foxp^high^ TILs; HR = 0.711; 95% CI, 0.381–1.101; *P* = 0.017) were independent prognostic factors for DFS in *EGFR*-mutant lung adenocarcinoma.

## 4. Discussion

The TME refers to the cellular environment in which tumors or cancer stem cells exist. Cancer stem cells are cells in a tumor with the abilities to self-renew and drive tumorigenesis [12]. The TME encompasses the surrounding immune cells, blood vessels, extracellular matrix (ECM), fibroblasts, lymphocytes, bone marrow-derived inflammatory cells, and signaling molecules [13, 14]. Interactions between malignant and nonmalignant cells create a TME that affects cancer development and progression [15, 16]. The nonmalignant cells in the TME often play a protumorigenic function at all phases of carcinogenesis by stimulating uncontrolled cell proliferation [17]. It has been reported that cancer development and progression are influenced by components of the TME and controlled by the host immune system [18]. Therefore, TME components and immune system biomarkers are important for cancer detection and evaluations of prognoses and treatment response [19]. The examination of the immune TME has a critical prognostic value and can supplement histopathological and molecular biomarkers with regard to the evaluation of patient responses to treatment. It is of great significance to further study the molecular mechanisms affecting TME.

Mechanistically, it has been well documented that PD-L1 expressed on tumor cells would facilitate tumor immune tolerance and evasion of the host by interacting with its receptor PD-1 on T cells and leading to T cell inactivation or exhaustion in the tumor microenvironment [20]. PD-L1 has been traditionally considered as a negative costimulatory molecule promoted constitutively by oncogenic driver mutations and indicates defective adaptive immune response in many solid tumors [21]. Following this rationale, the overexpression of PD-L1 by tumor cells behooves to correlate with poorer prognosis [22]. However, according to our findings, *EGFR*-mutant tumors have higher expression of PD-L1 than tumors without *EGFR* mutations. And PD-1 expression levels appear to be negative correlated. The results contradict some previous studies [23]. This result may be due to the small sample size of this study, which is insufficient to explain the expression correlation between them. Actually, the diversity remains in the outcome obtained from different studies investigating whether PD-L1 could be recognized as an effective biomarker of prognosis. Maybe, high PD-L1 expression in *EGFR*-mutant patients may be only a result of the oncogene drive, rather than the main cause of tumor immune escape [24].

The immune infiltrate, in lung cancers as well as in other malignancies, has been shown to comprise adaptive and innate immune cells [25]. Here, we evaluated the infiltration of lung adenocarcinoma CD3^+^ T cell, CD4^+^ T cell, CD8^+^ T cell, Foxp3^+^ T cell, and their matched normal tissues. Our study showed that tumor-infiltrating Treg cells are different from normal tissue-infiltrating Tregs, suggesting that the tumor microenvironment influences specific gene expression in Treg cells, and further support the view that Treg cells from different tissues are instructed by environmental factors to display different gene expression profiles. These data could help a better understanding of the Treg functional role at tumor sites and pave the way to the identification of therapeutic targets for more specific and safer modulation of Treg cells in cancer therapy.

We took the univariate and multivariate analyses to find factors that contribute to DFS in lung adenocarcinoma. Increased Treg frequency has been associated with poor outcomes in cancer patients [26, 27]. Our results showed that high infiltration of Tregs in our cohorts was positively associated with poor prognosis of patients. The frequency of Tregs in tumors with *EGFR* mutations was significantly higher than that in wild-type tumors. The regulatory T cells (Tregs) are one of the most important inhibitory components in the TME [28]; Tregs influence the tumor microenvironment during the progression of lung cancers [29]. Murine models of lung adenocarcinoma have demonstrated that Tregs may inhibit CD8 T cell-mediated antitumor immunity, with the depletion of Tregs resulting in tumor cell death and elevated levels of granzyme A, granzyme B, perforin, and IFN-*γ* in infiltrating CD8 T cells at early stages of tumorigenesis [30]. Further, the development of SCLC influences immunosuppressive activities of Tregs, where SCLC cell lines were reported to induce Treg generation from CD4 T cells through the production of IL-15 [31]. A clinical study of NSCLC observed that Treg levels in peripheral blood increased with the stage and were highest in patients with metastatic tumors [32]. Emerging evidence suggests that Tregs promote metastasis and metastatic tumor focus development [33]. Other factors are at play and the molecular mechanisms underlying Treg recruitment and their immunosuppressive functions in the lung tumor microenvironment require further study to improve patient therapy and outcomes.

PD-L1 expression on tumor cells could be attributed to IFN-*γ* production by TILs, which is in association with powerful antitumor immunity and favorable prognosis in theory [17]. Prognostically, a high proportion of Foxp3 lymphocytes in SCLC lung tumor biopsies correlates with poor survival [18]. Another study in NSCLC identified that elevated levels of intertumoral Foxp3 lymphocytes were associated with reduced recurrence-free survival [19]. In the study, we found that patients with age ≥ 66 years and Foxp^low^ TILs were independent prognostic factors for DFS in EGFR-mutant lung adenocarcinoma. This finding may provide a novel option for prognosis prediction of lung adenocarcinoma patients.

It must be acknowledged that our research has several limitations. First, the EGFR wild-type group may have been heterogeneous, leading to the possible inclusion of patients with other mutations (KRAS, TP53, ROS1, STK11, etc.), which may influence PD-L1 expression and CD8^+^ T cell infiltration. Second, all the patients were in the early stage and the data of OS was not available. Third, there is a lack of in-depth analysis of the immune landscape, including proliferation T cells, effector T cell, naive T cells, NK, DC, B cells, and MDSC. Therefore, we intend to further analyze subsequent single-cell sequencing.

In summary, our study is the first to clarify the detailed differences in immunological landscape in lung adenocarcinoma with and without *EGFR* mutation. Our findings offer novel evidence that *EGFR*-mutant tumors have a higher infiltration of Tregs compared with wild-type tumors, which could be the main cause of impaired response to PD-1 pathway blockade in *EGFR*-driven lung adenocarcinoma. Besides, our research suggests that Tregs could be regarded as a promising target for treatment of lung adenocarcinoma in the future.

## Figures and Tables

**Table 1:** 

Factor	Category	Mutant		Wild type	
		No. of patients	%	No. of patients	%
Age (years)	Median (range)	67.3 (41–86)		64.8 (35–86)	

Gender	Male	99	60.4	114	71.7
	Female	65	39.6	45	28.3

Smoking status	Never smoker	147	89.6	144	90.6
	Smoker	17	10.4	15	9.5

T status	T1	80	48.8	100	62.9
	T2	79	48.2	55	34.6
	T3	5	3	4	2.5

N status	N0	118	72	115	72.3
	N1	30	18.3	31	19.5
	N2	16	9.8	13	8.2

p-stage	I	90	54.9	84	52.8
	II	51	31.1	48	30.2
	IIIa	23	14	27	17

VI	Positive	31	18.9	28	17.6
	Negative	133	81.1	131	82.4

VPSI	Positive	101	61.6	103	64.8
	Negative	63	38.4	56	35.2

Surgery procedure	Lobectomy + wedge	148	90.2	141	88.7
	Pneumonectomy	16	9.8	18	11.3

**Table 2:** 

		EGFR mutant					Wild type			
		Univariate	*P* value	HR	Multivariate	*P* value	Univariate	*P* value	HR	Multivariate	
Clinicopathologic features	HR	95% CI			95% CI		HR95% CI			95% CI*P* value	
Age ≥ 66 vs <66	1.305	0.988 to 2.987	0.030	1.169	1.112 to 1.877	**0.009**	1.1430.329 to 2.619	0.811			
Male vs female	0.927	0.539 to 2.101	0.893				1.6240.510 to 4.364	0.351			
Smoking vs no smoking	1.323	0.321 to 3.257	0.610				1.1120.211 to 3.401	0.941			
T stage: T1 vs T2–3	0.391	0.145 to 0.840	0.021	0.531	0.126 to 1.216	0.323	0.7180.208 to 1.204	0.513			
N stage: N0 vs N1–2	0.801	0.354 to 2.135	0.611				0.1640.043 to 0.491	0.001	0.312	0.089 to 1.1210.069	
Pathological stage: I vs II–IIIA	0.331	0.164 to 0.805	0.022	0.421	0.170 to 1.214	0.132	0.2270.079 to 0.799	0.014	0.316	0.075 to 2.1990.314	
Vascular infiltration: positive vs negative	3.788	1.573 to 15.201	0.001	0.601	0.283 to 1.729	0.301	3.2011.609 to 10.821	0.004	0.716	0.266 to 2.2150.715	
Pleural infiltration: positive vs negative	3.804	1.277to 7.112	0.016	0.721	0.219 to 2.228	0.739	0.8860.367 to 2.112	0.801			
Surgical method: wedge resection + lobectomy vs total resection	0.704	0.195 to 2.140	0.622				2.7210.518 to 6.412	0.301			
CD3: low vs high	1.311	0.538 to 2.106	0.434				0.7320.236 to 2.112	0.615			
CD4: low vs high	1.201	0.501 to 2.135	0.501				1.1330.368 to 2.160	0.829			
CD8: low vs high	0.820	0.417 to 2.007	0.277				1.2440.442 to 3.223	0.544			
Foxp3: low vs high	0.713	0.289 to 0.912	0.022	0.711	0.381 to 1.101	**0.017**	2.1360.711 to 4.111	0.201			
LAG3: low vs high	2.014	0.834 to 4.324	0.112				4.2421.609 to 6.757	0.126			
PD-L1: positive vs negative	1.114	0.492 to 2.303	0.791				0.6500.213 to 2.406	0.611			
PD-1: low vs high	0.335	0.654 to 1.142	0.031	0.402	0.413 to 1.212	0.102	0.6990.211 to 2.112	0.664			

## Data Availability

No data were used to support this study.
